# Clinical significance and efficacy of endoscopic ultrasound‐guided tissue acquisition for para‐aortic lymph node metastasis

**DOI:** 10.1111/den.15009

**Published:** 2025-03-10

**Authors:** Hidenobu Hara, Susumu Hijioka, Daiki Yamashige, Yoshikuni Nagashio, Yasuhiro Komori, Masaru Kuwada, Soma Fukuda, Shin Yagi, Kohei Okamoto, Daiki Agarie, Mark Chatto, Mao Okada, Yuta Maruki, Chigusa Morizane, Hideki Ueno, Yutaka Saito, Kan Yonemori, Takuji Okusaka

**Affiliations:** ^1^ Department of Hepatobiliary and Pancreatic Oncology National Cancer Center Tokyo Japan; ^2^ Cancer Medicine Jikei University Graduate School of Medicine Tokyo Japan; ^3^ Endoscopy Division National Cancer Center Tokyo Japan; ^4^ Department of Breast and Medical Oncology National Cancer Center Tokyo Japan; ^5^ Department of Gastroenterology Makati Medical Center Makati City Philippines

**Keywords:** accuracy, endoscopic ultrasound‐guided tissue acquisition, para‐aortic lymph node, resectable pancreatic cancer

## Abstract

**Objectives:**

Assessing para‐aortic lymph node (PALN) metastasis in solid tumors is crucial for accurate staging. In clinical practice, PALN metastasis is typically diagnosed based on imaging findings; however, the efficacy of endoscopic ultrasound‐guided tissue acquisition (EUS‐TA) in diagnosing PALN metastasis remains insufficiently understood.

**Methods:**

This single‐center, retrospective study included patients who underwent EUS‐TA of PALNs and computed tomography (CT). Final diagnoses were based on pathological findings or 12‐month imaging follow‐up.

**Results:**

Among 167 patients, technical success was achieved in 162 (97.0%). EUS‐TA demonstrated a sensitivity, specificity, and accuracy of 85.1% (63/74), 100% (88/88), and 93.2% (151/162), respectively, for PALN metastasis. These results showed significantly higher sensitivity (28.4% vs. 85.1%, *P* < 0.001) and accuracy (64.8% vs. 93.2%, *P* < 0.001) than those of CT. The accuracy of CT and EUS‐TA was 86.8% vs. 89.5% for PALNs measuring <5 mm, 51.5% vs. 92.9% for those measuring 5–10 mm, and 84.0% vs. 96.0% for those measuring ≥10 mm, with a significant difference in the 5–10 mm category (*P* < 0.001). Among the 44 patients diagnosed with resectable pancreatic cancer using CT, the final diagnosis confirmed PALN metastasis in 10 (22.7%) patients, and EUS‐TA preoperatively identified PALN metastasis in eight (18.2%) patients. EUS‐TA significantly reduced unnecessary surgeries compared with CT‐only diagnoses (*P* = 0.013).

**Conclusion:**

Endoscopic ultrasound‐guided tissue acquisition of PALNs offers high diagnostic accuracy and can detect PALN metastasis often missed by CT alone. Integrating EUS‐TA into preoperative assessments has the potential to substantially reduce unnecessary surgeries, improve patient outcomes, and plan treatment strategies.

## INTRODUCTION

Para‐aortic lymph node (PALN) metastasis, like liver and lung metastases, indicates distant metastasis and is considered inoperable. PALN assessment is crucial for cancer staging diagnoses. Particularly, in solid tumors considered potentially resectable via preoperative assessment, an accurate diagnosis of PALN metastasis is critical for determining the treatment approach. However, imaging modalities, primarily computed tomography (CT), have limited sensitivities ranging from 21% to 60%, which is considered insufficient.^1^, ^2^, ^3^ Specifically, in pancreatic cancer (PC), sensitivity, specificity, and accuracy are 21%, 86%, and 75% for CT, 21.4%, 93.3%, and 76.3% for magnetic resonance imaging, and 7.7%, 94.2%, and 96.2% for endoscopic ultrasonography (EUS), respectively. Hence, imaging alone is insufficient for preoperatively assessing PALNs.^4^

These limitations can lead to misclassification of resectability, resulting in unnecessary surgeries or inappropriate treatment plans. Indeed, 12–20% of patients with PC considered operable by imaging present with PALN metastasis at the time of surgery.^3^, ^5^, ^6^, ^7^ An accurate preoperative diagnosis of PALN metastasis can prevent unnecessary surgery and facilitate early pharmacological treatment. Thus, there is an urgent need to improve the current diagnostic capabilities for PALN metastasis.

Nevertheless, research on alternative methods, such as endoscopic ultrasound‐guided tissue acquisition (EUS‐TA), remains limited.^8^ Given the technical difficulties and scarcity of studies on applying EUS‐TA to detect PALN metastasis, many unresolved issues remain. To our knowledge, no studies have compared the diagnostic capabilities of CT and EUS‐TA for PALN metastasis. Therefore, this study aimed to fill these gaps by evaluating the efficacy, safety, and clinical significance of EUS‐TA in diagnosing PALN metastasis, potentially solving the limitations of conventional imaging.

## METHODS

### Patients and data collection

**Figure 1:**
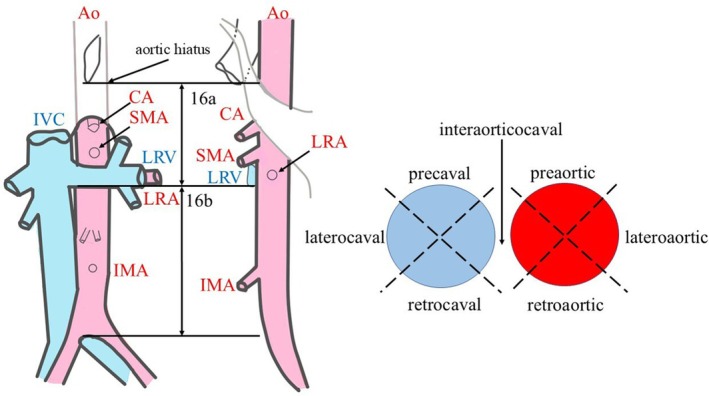
Schematic of the location of para‐aortic lymph nodes. Ao, aorta; CA, celiac artery; IMA, inferior mesenteric artery; IVC, inferior vena cava; LRA, left renal artery; LRV, left renal vein; SMA, superior mesenteric artery. 16a, from the aortic hiatus to the inferior border of the left renal vein; 16b, inferior border of the left renal vein to the abdominal aortic bifurcation.

This single‐center, retrospective study was conducted between October 2017 and March 2024. The inclusion criteria were that the primary disease was suspected of malignancy on CT and patients were enrolled for EUS‐TA of PALNs. Patients with benign primary disease, lymphoma, or those having difficulty with a final diagnosis of PALN metastasis, were excluded. Data regarding age, sex, PALN localization and size, puncture site, number of punctures, and primary diagnosis were collected from databases and medical records. PALN localization and size were determined by CT (Fig. 1). PALNs identified by EUS were confirmed through re‐evaluation of CT images. Imaging was primarily performed using a CT system (Aquilion Precision; Canon Medical Systems, Ohtawara, Japan). The size of PALNs was measured on plain CT with a slice thickness of 2 mm. PC staging was based on dynamic CT findings using the contrast agent Oypalomin 300 (Fuji Pharma, Tokyo, Japan). Written informed consent was obtained from all patients, and this study was approved by the Ethics Committee of the National Cancer Center (approval number: 2018‐149).

### EUS‐TA procedure

**Figure 2:**
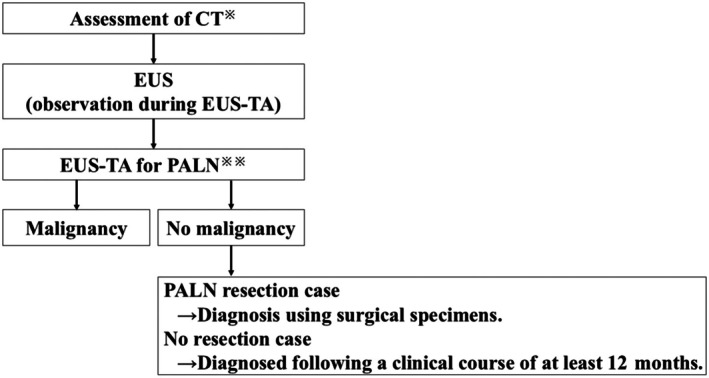
Procedure flowchart. CT, computed tomography; EUS, endoscopic ultrasound; EUS‐TA, endoscopic ultrasound‐guided tissue acquisition; PALN, para‐aortic lymph node. 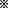, Short axis of 10 mm or more is considered a malignancy; 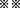, technical success is defined as the successful puncture achieved by EUS.

**Figure 3:**
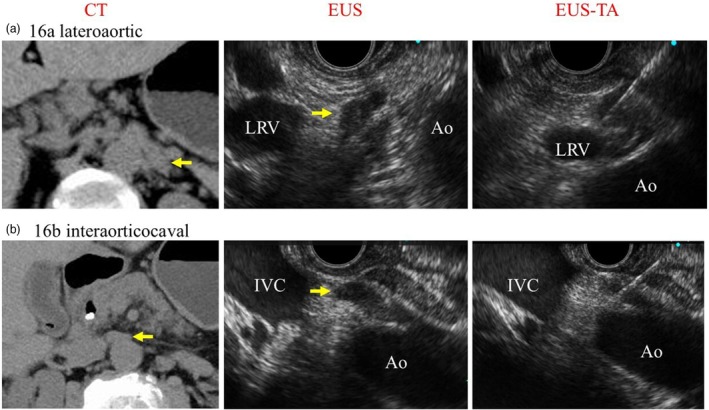
Computed tomograph (CT) and endoscopic ultrasound‐guided tissue acquisition (EUS‐TA) images of para‐aortic lymph nodes. Ao, aorta; EUS, endoscopic ultrasound; IVC, inferior vena cava; LRV, left renal vein. (a) 16a lateroaortic observation from the stomach. (b) 16b interaorticocaval observation from the descending segment.

Endoscopy used a convex EUS scope (GF‐UCT260; Olympus, Tokyo, Japan, or EG‐580UT; Fujifilm, Tokyo, Japan). PALNs were observed concurrently during the EUS‐TA of the primary lesion to assess whether PALNs were visible and suitable for puncture (Fig. 2). The preferred approach for lateroaortic lesions was puncture from the stomach, whereas for interaorticocaval lesions, puncture from the duodenum was the first choice (Fig. 3). If vessel interference made puncture difficult, observations were conducted from various sites to determine a feasible puncture site. Trainees or trainers performed the procedures under the supervision or partial assistance of an endoscopist experienced in over 500 EUS‐TA procedures to ensure safety and quality.

The needle was primarily a 22G needle; selecting between a fine‐needle aspiration (FNA) or fine‐needle biopsy (FNB) was based on the purpose of the examination. For specimen collection, the slow pull technique was used with an FNB and 20 mL syringe negative pressure with an FNA. Typically, 20 strokes were performed per session, and a portion of the sample was used for rapid on‐site evaluation with Diff–Quik staining (Sysmex Corporation, Kobe, HG, Japan).

### Definitions

Based on the assessment of PALNs using plain CT before performing EUS‐TA, the localization was categorized according to the Japanese Classification of Pancreatic Carcinoma (Fig. 1).^9^ Technical success was defined as the visualization of the needle tip within PALN during EUS observation. Cases where puncture of PALN was made possible by changing the needle were also defined as a technical success. The classification of EUS features was based on a previous report.^10^

**Figure 4:**
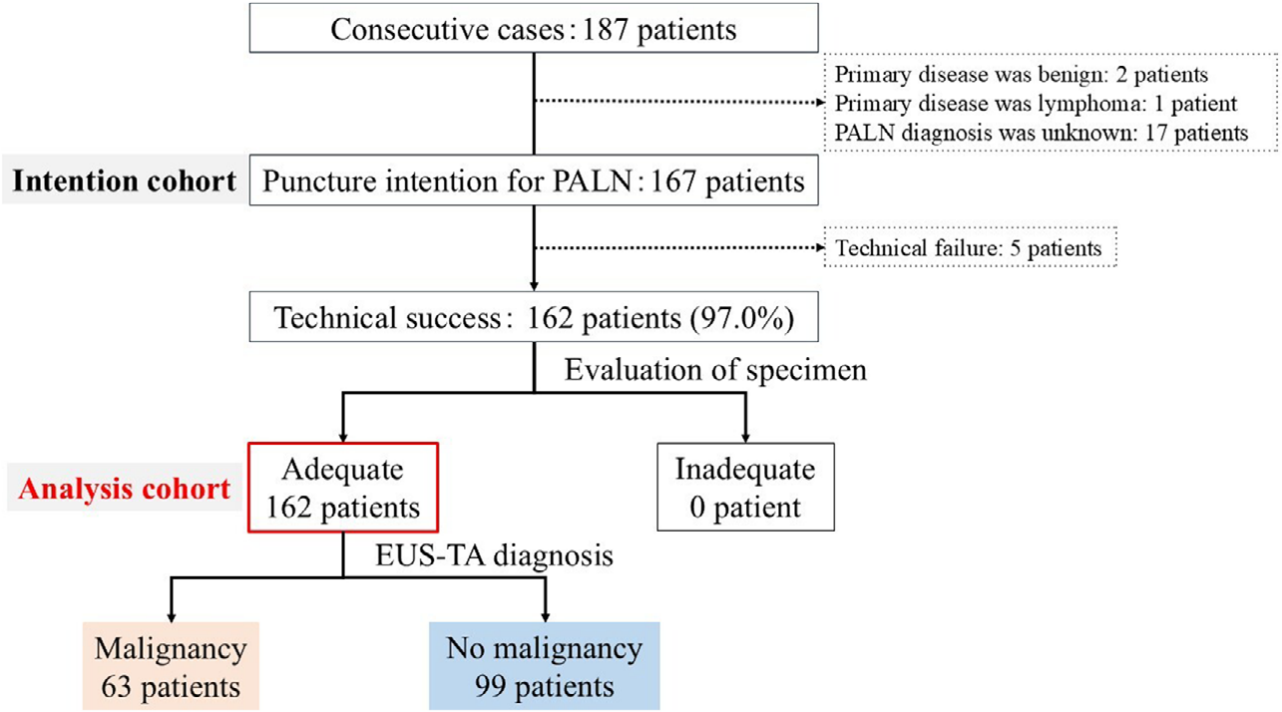
Flow diagram of the study. EUS‐TA, endoscopic ultrasound‐guided tissue acquisition; PALN, para‐aortic lymph node.

The analysis cohort included patients in whom technical success was achieved, and whose EUS‐TA specimens were deemed adequate for diagnosis by the pathologist (Fig. 4).^11^ Adverse events were evaluated using the severity grading system of the American Society for Gastrointestinal Endoscopy Lexicon classification.^12^ When multiple PALNs were punctured, either the PALN diagnosed as malignant at the final diagnosis or the one with the largest short axis was analyzed. Previous studies on PALN have not established objective diagnostic criteria using contrast agents, and diagnostic inconsistencies have been reported among radiologists.^13^, ^14^, ^15^ Therefore, this study objectively evaluated PALN metastasis based on the short axis in plain CT. According to the Japanese Classification of Pancreatic Carcinoma^9^ and RECIST version 1.1,^16^ lymph nodes with a short axis of ≥10 mm were considered metastatic. This study diagnosed PALN with the short axis ≥10 mm on plain CT as the metastasis. Based on previous reports, the optimal standardized uptake value maximum (SUVmax) cut‐off value for positron emission tomography (PET)‐CT to diagnose metastases was set at 1.8.^8^

The final diagnosis of a PALN was classified as follows (Fig. 2):

- Pathological diagnosis of malignancy by EUS‐TA.
- Pathological diagnosis of no malignancy by EUS‐TA.

For patients who underwent surgical resection, the final diagnosis was based on a pathological diagnosis of a PALN in the resected specimen.

When surgical resection confirmed PALN metastasis and EUS‐TA showed false‐negative findings, lymph node pathology was reviewed and classified into four categories based on tumor percentage within PALN: <25%, 25–50%, 50–75%, and >75%.

For patients who did not undergo resection of PALNs, an imaging follow‐up examination was conducted at 12 months, and no change in PALN size indicated absence of malignancy. Imaging diagnosis was performed by radiologists with board certifications in radiology.

The final pathological diagnosis was confirmed as malignancy if the histopathological tissue examination revealed malignancy or the cytological examination indicated Papanicolaou class 4/5.

### Outcome

The primary outcome was accuracy of EUS‐TA. The secondary outcome included the technical success rate of EUS‐TA, adverse event rate, comparison of accuracy between CT and EUS‐TA, accuracy of EUS‐TA by PALN size, and proportion of resectable PC cases where the treatment strategy was altered based on EUS‐TA findings.

### Statistical analyses

Continuous variables are presented as medians and interquartile ranges (IQRs), and categorical variables as numbers and proportions. Confidence intervals (CIs) were calculated using an exact binomial distribution. Diagnostic methods were compared using McNemar, Fisher's exact, or Cochran's *Q* tests. Two‐sided *P*‐values of <0.05 were considered statistically significant. All analyses were performed using SPSS (version 27.0; IBM, Armonk, NY, USA).

## RESULTS

Endoscopic ultrasound‐guided tissue acquisition was performed in 167 patients, with a technical success rate of 97.0% (162/167). All technically successful cases were assessed as having adequate specimens (Fig. 4).

The characteristics of 162 patients in the analysis cohort are shown in Table 1. The median age (IQR) was 71 (61–77) years. EUS‐TA was performed to diagnose primary malignant lesions in 150 patients. The diagnosis of the primary disease was PC in 81 (resectable in 44) patients and biliary tract cancer in 56.

**Table 1:** Patient characteristics^†^

Analysis cohort	Patients (*N* = 162)
Age, years	71 (61–77)
Sex, male	80 (49.4)
Purpose of EUS‐TA	
Diagnosis of primary disease	150 (92.6)
Diagnosis of recurrence	12 (7.4)
Primary disease	
Pancreatic cancer	81 (50.0)
R/BR/UR	44 (27.2)/19 (11.7)/18 (11.1)
Biliary tract cancer	56 (34.6)
Neuroendocrine neoplasm	12 (7.4)
Gastrointestinal tract cancer	5 (3.1)
Cancer of unknown primary	3 (1.9)
Breast cancer	1 (0.6)
Ovarian cancer	1 (0.6)
Renal cell cancer	1 (0.6)
Melanoma	1 (0.6)
Seminoma	1 (0.6)

^†^ Data are presented as *n* (%) or median (interquartile range).

BR, borderline resectable pancreatic cancer; EUS‐TA, endoscopic ultrasound‐guided tissue acquisition; R, resectable pancreatic cancer; UR, unresectable pancreatic cancer.

Details of PALNs and EUS‐TA techniques are shown in Table 2. The median long and short axes (IQR) of PALNs on CT were 11.3 (8.7–14.8) mm and 6.7 (5.0–8.7) mm, respectively. In 143 patients, only one lesion was punctured. The main locations of PALNs were 16b interaorticocaval in 74 and 16b lateroaortic in 52.

**Table 2:** Detailed characteristics of para‐aortic lymph nodes (PALNs) and procedural outcomes of endoscopic ultrasound‐guided tissue acquisition (EUS‐TA)^†^

Analysis cohort	Patients (*N* = 162)
PALN information	
Diameter of PALN	
Long axis, mm	11.3 (8.7–14.8)
Short axis, mm	6.7 (5.0–8.7)
Lesions planned for puncture	
One lesion/two lesions	143 (88.3)/19 (11.7)
Location of PALN	
16b interaorticocaval	74 (45.7)
16b lateroaortic	52 (32.1)
16a lateroaortic	27 (16.7)
16a interaorticocaval	7 (4.3)
16b preaortic	2 (1.2)
EUS‐TA procedure	
Technical success	162 (97.0)
Puncture site	
Transgastric/transduodenal	79 (48.8)/83 (51.2)
Needle size	
19/22/25G	4 (2.5)/156 (96.3)/2 (1.2)
FNA/FNB	123 (75.9)/39 (24.1)
Change from FNB to FNA	6 (3.7)
Median number of punctures	2 (2–3)
Adequate specimen	162 (100)
Adverse event	1 (0.6)

^†^ Data are presented as *n* (%) or median (interquartile range).

FNA, fine‐needle aspiration; FNB, fine‐needle biopsy.

In all, 79 patients underwent a puncture from the stomach. A 22G needle was used in 156 patients. FNA was used in 123 patients and FNB in 39 (Table S1). In six patients, the needle type was changed from FNB to FNA due to difficulties in puncture. The median number of punctures (IQR) was 2 (2–3). The rate of obtaining an adequate pathological specimen was 100%. Adverse events were observed in one patient (0.6%) who developed an intra‐abdominal abscess (grade: severe) following a puncture with a 19G FNB needle.

### Diagnostic capability of EUS‐TA for PALN metastasis

The diagnostic results of EUS‐TA for PALN are presented in Table 3. EUS‐TA diagnosed 63 patients with malignancy and 99 patients with no malignancy. Final diagnoses were malignancy in 74 patients and no malignancy in 88. The diagnostic capabilities of EUS‐TA were as follows: sensitivity, 85.1% (63/74); specificity, 100% (88/88); positive predictive value, 100% (63/63); negative predictive value (NPV), 88.9% (88/99); and accuracy, 93.2% (151/162) (Table 4). Among the cases diagnosed as no malignancy by EUS‐TA, 11.1% (11/99) were false‐negatives. Among the false‐negatives, 63.6% (7/11) had a short axis of PALNs measuring 5–10 mm, with EUS findings showing sharp borders in nine cases, and findings consistent with metastasis across all parameters in four cases (Table 5). Three cases were malignant based on the resected specimen, including one where the PALN targeted by EUS‐TA was different from the one removed (Table 5, Case No. 8). Additionally, eight cases became unresectable due to primary tumor progression, with PALN enlargement identified and subsequently diagnosed as malignant.

**Table 3:** Diagnostic yield of endoscopic ultrasound‐guided tissue acquisition (EUS‐TA) for para‐aortic lymph nodes

	Final diagnosis		Total
	Malignancy	No malignancy	
EUS‐TA			
Malignancy	63	0	63
No malignancy	11	88	99
Total	74	88	162

**Table 4:** Diagnostic yields of endoscopic ultrasound‐guided tissue acquisition in the analysis cohort and patients with resectable pancreatic cancer

	Diagnostic yield, % (95% CI)	
	Analysis cohort (*N* = 162)	Resectable pancreatic cancer (*n* = 44)
Sensitivity	85.1 (75.0–92.3)	80.0 (44.4–97.5)
Specificity	100 (93.9–100)	100 (85.1–100)
PPV	100 (91.6–100)	100 (51.8–100)
NPV	88.9 (81.0–94.3)	94.4 (81.3–99.3)
Accuracy	93.2 (88.2–96.6)	95.5 (84.5–99.4)

Values are presented as percentage (95% confidence interval [CI]).

EUS‐TA, endoscopic ultrasound‐guided tissue acquisition; NPV, negative predictive value; PALN, para‐aortic lymph node; PPV, positive predictive value.

**Table 5:** All cases of misdiagnosis by endoscopic ultrasound‐guided tissue acquisition (EUS‐TA)

Case No.	Primary disease	Long/short axis (mm)	Puncture needle/gauge	Number of punctures	Lesion	Diagnosis of EUS‐TA	Final diagnosis (method)	FN/FP	Resected lesion	Tumor proportion in PALN	EUS features
1	Pancreatic cancer	8.8/4.4	FNA/22G	1	16b lateroaortic	No malignancy	FU	FN	–	–	Ellipsoid, sharp, heterogeneous, hyperechoic
2	Pancreatic cancer	10.9/4.9	FNB/22G	2	16a lateroaortic	No malignancy	FU	FN	–	–	Ellipsoid, sharp, heterogeneous, hyperechoic
3	Pancreatic cancer	7.3/5.0	FNA/22G	2	16b lateroaortic	No malignancy	FU	FN	–	–	Round, sharp, heterogeneous, hyperechoic
4	Pancreatic cancer	8.5/7.0	FNA/22G	2	16b lateroaortic	No malignancy	FU	FN	–	–	Round, sharp, homogeneous, hypoechoic
5	Pancreatic cancer	13.3/7.4	FNB/22G	3	16b interaorticocaval	No malignancy	Resection	FN	16b interaorticocaval	<25%	Round, sharp, homogeneous, hypoechoic
6	Pancreatic cancer	19.7/9.6	FNB/22G	2	16b interaorticocaval	No malignancy	FU	FN	–	–	Round, fuzzy, homogeneous, hypoechoic
7	Pancreatic cancer	27.0/17.3	FNB/22G	1	16b lateroaortic	No malignancy	FU	FN	–	–	Round, sharp, homogeneous, hypoechoic
8	Biliary tract cancer	8.3/3.1	FNA/22G	2	16b lateroaortic	No malignancy	Resection	FN	16b interaorticocaval	<25%	Ellipsoid, sharp, homogeneous, hypoechoic
9	Biliary tract cancer	17.4/7.1	FNB/22G	3	16b interaorticocaval	No malignancy	Resection	FN	16b interaorticocaval	<25%	Ellipsoid, fuzzy, heterogeneous, hyperechoic
10	Biliary tract cancer	10.3/9.3	FNA/22G	2	16b lateroaortic	No malignancy	FU	FN	–	–	Round, sharp, homogeneous, hypoechoic
11	Unknown primary	15.2/6.2	FNA/22G	2	16b interaorticocaval	No malignancy	FU	FN	–	–	Ellipsoid, sharp, heterogeneous, hyperechoic

EUS, endoscopic ultrasound; FN, false‐negative; FNA, fine‐needle aspiration; FNB, fine‐needle biopsy; FP, false‐positive; FU, increase by follow‐up; PALN, para‐aortic lymph node.

**Figure 5:**
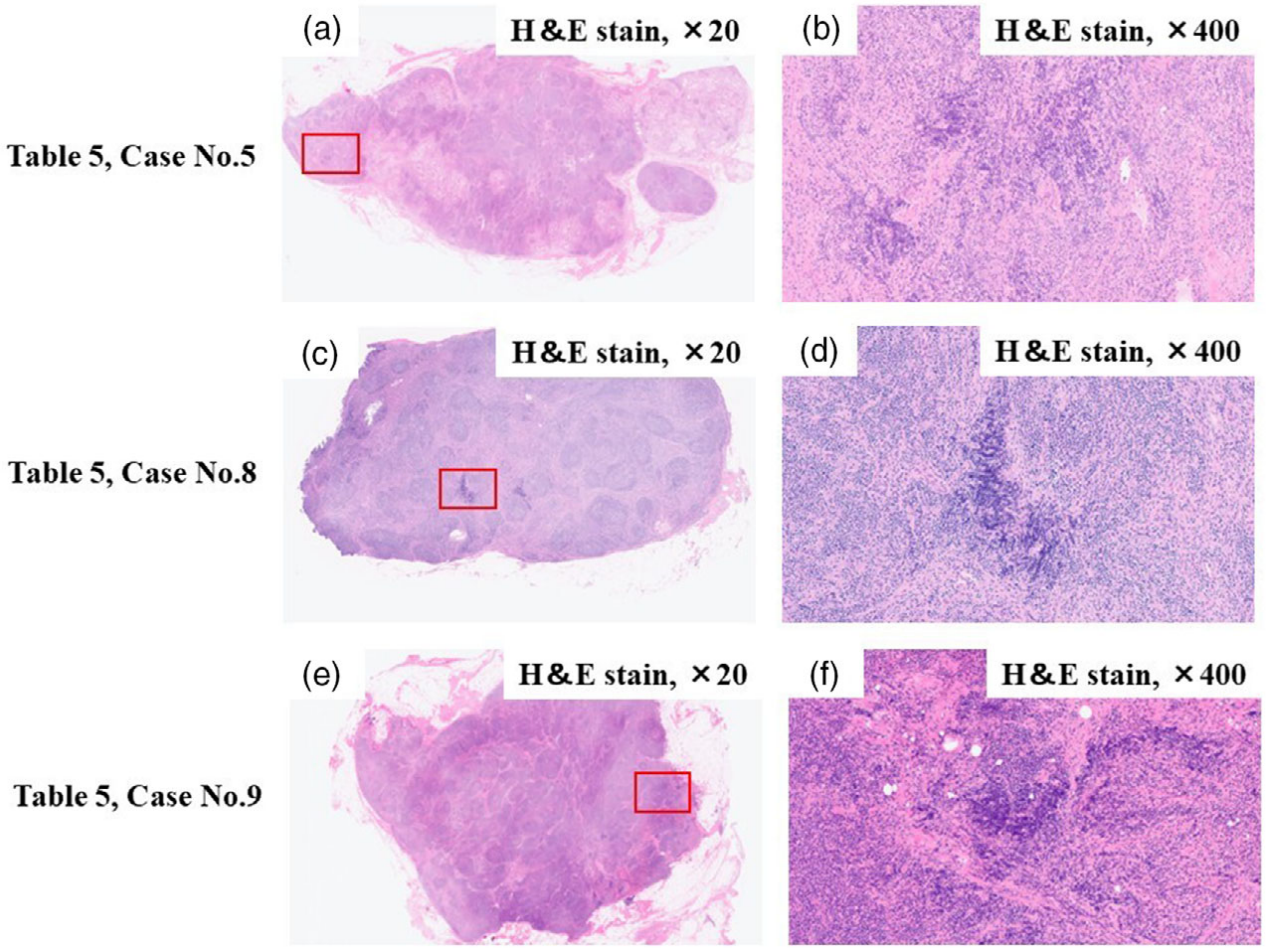
Pathological findings of surgically resected para‐aortic lymph node (PALN) in patients with false‐negative findings on endoscopic ultrasound‐guided tissue acquisition. (a,b) Images present a case of pancreatic cancer (Table 5, Case No. 5). (c,d) Images present a case of biliary tract cancer (Table 5, Case No. 8). (e,f) Images present a case of biliary tract cancer (Table 5, Case No. 9). The lymphoid follicles are well preserved, with lymphocyte infiltration observed at the margins. PALN metastasis is observed in part of the resected specimen (square, ×400). H&E, hematoxylin and eosin.

For the three surgically identified false‐negative cases, the tumor proportion within the PALNs was evaluated using the defined criteria. All three cases showed tumor proportions of <25% (Table 5). Tumors were observed at the margins and showed a patchy distribution throughout the PALN (Fig. 5).

The diagnostic capabilities of FNA and FNB are shown in Table 6, with no significant difference observed in sensitivity (86.7% vs. 82.8%, *P* = 0.742) or accuracy (95.1% vs. 87.2%, *P* = 0.136).

**Table 6:** Comparison of fine‐needle aspiration (FNA) and fine‐needle biopsy (FNB) needles

	Analysis cohort, % (95% CI)		*P*‐value*
	FNA (*n* = 123)	FNB (*n* = 39)	
Sensitivity	86.7 (73.2–94.9)	82.8 (64.2–94.2)	0.742
Specificity	100 (93.1–100)	100 (58.7–100)	1.000
PPV	100 (86.8–100)	100 (79.6–100)	1.000
NPV	92.9 (85.1–97.3)	75.0 (38.4–88.2)	0.011
Accuracy	95.1 (89.7–98.2)	87.2 (72.6–95.7)	0.136

Values are presented as percentage (95% confidence interval [CI]).

* Fisher's test *P*‐value.

FNB, fine‐needle biopsy; NPV, negative predictive value; PPV, positive predictive value.

### Comparison of the diagnostic accuracy between CT and EUS‐TA for PALN metastasis

**Figure 6:**
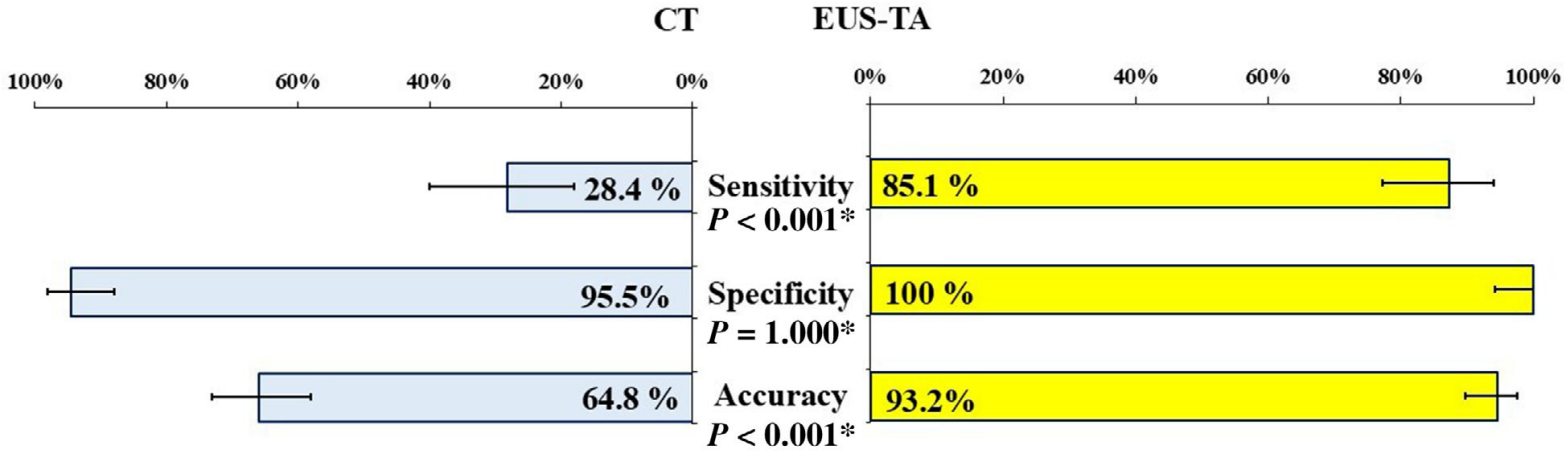
Comparative diagnostic yield between computed tomography (CT) and endoscopic ultrasound‐guided tissue acquisition (EUS‐TA). The figure shows diagnostic yields and 95% confidence intervals. The diagnostic yield of EUS‐TA was significantly higher than that of CT in terms of sensitivity and accuracy, as assessed by the McNemar test. *McNemar test.

The diagnostic capabilities of CT vs. EUS‐TA are depicted in Figure 6. Sensitivity, specificity, and accuracy of CT were 28.4% (21/74), 95.5% (84/88), and 64.8% (105/162), respectively. EUS‐TA demonstrated significantly higher sensitivity and accuracy (sensitivity, *P* < 0.001; accuracy, *P* < 0.001) than CT. The NPV of EUS‐TA (88.9%, 88/99) was also superior to CT (61.3%, 84/137).

Positron emission tomography‐computed tomography (PET‐CT) was performed in 32 patients (19.8%) and had an accuracy of 75.0%. EUS‐TA demonstrated a higher accuracy of 87.5%; however, the difference was not statistically significant (*P* = 0.343; Table S2).

The diagnostic accuracy of CT and EUS‐TA for PALN size is presented in Table 7. For PALNs measuring <5 mm, the malignancy rate was 13.2%, with no significant difference in accuracy between CT and EUS‐TA (86.8% vs. 89.5%, *P* = 0.480). For PALNs measuring 5–10 mm, the malignancy rate was 48.5%, and a significant difference in the diagnostic accuracy was observed between CT and EUS‐TA (51.5% vs. 92.9%, *P* < 0.001). For PALNs measuring ≥10 mm, the malignancy rate was 84.0%, with no significant difference in accuracy between CT and EUS‐TA (84.0% vs. 96.0%, *P* = 0.371). EUS‐TA consistently showed higher accuracy than CT across all size categories of PALNs.

**Table 7:** Accuracy of computed tomography (CT) and endoscopic ultrasound‐guided tissue acquisition (EUS‐TA) in measuring the short axis diameter of para‐aortic lymph nodes (PALNs)

Short axis diameter of PALN (mm)	Malignancy rate	Accuracy, % (95% CI)		*P*‐value*
		CT	EUS‐TA	
<5	13.2% (5/38)	86.8 (71.9–95.6)	89.5 (75.2–97.1)	0.480
**5–10**	**48.5% (48/99)**	**51.5 (41.3–61.7)**	**92.9 (86.0–97.1)**	**<0.001**
≥10	84.0% (21/25)	84.0 (63.9–95.5)	96.0 (79.6–99.9)	0.371
**Total**	**45.7% (74/162)**	**64.8 (56.9–72.1)**	**93.2 (88.2–96.6)**	**<0.001**

Values are expressed as percentage (95% confidence interval [CI]). Bold indicates statistical significance.

* McNemar test *P*‐value.

CI, confidence interval; CT, computed tomography; EUS‐TA, endoscopic ultrasound‐guided tissue acquisition; PALN, para‐aortic lymph node.

Among 32 patients who underwent PET‐CT, EUS‐TA consistently demonstrated superior diagnostic accuracy across all size categories (Table S3).

### Impact of EUS‐TA on the treatment strategy for CT‐diagnosed resectable pancreatic cancer

**Figure 7:**
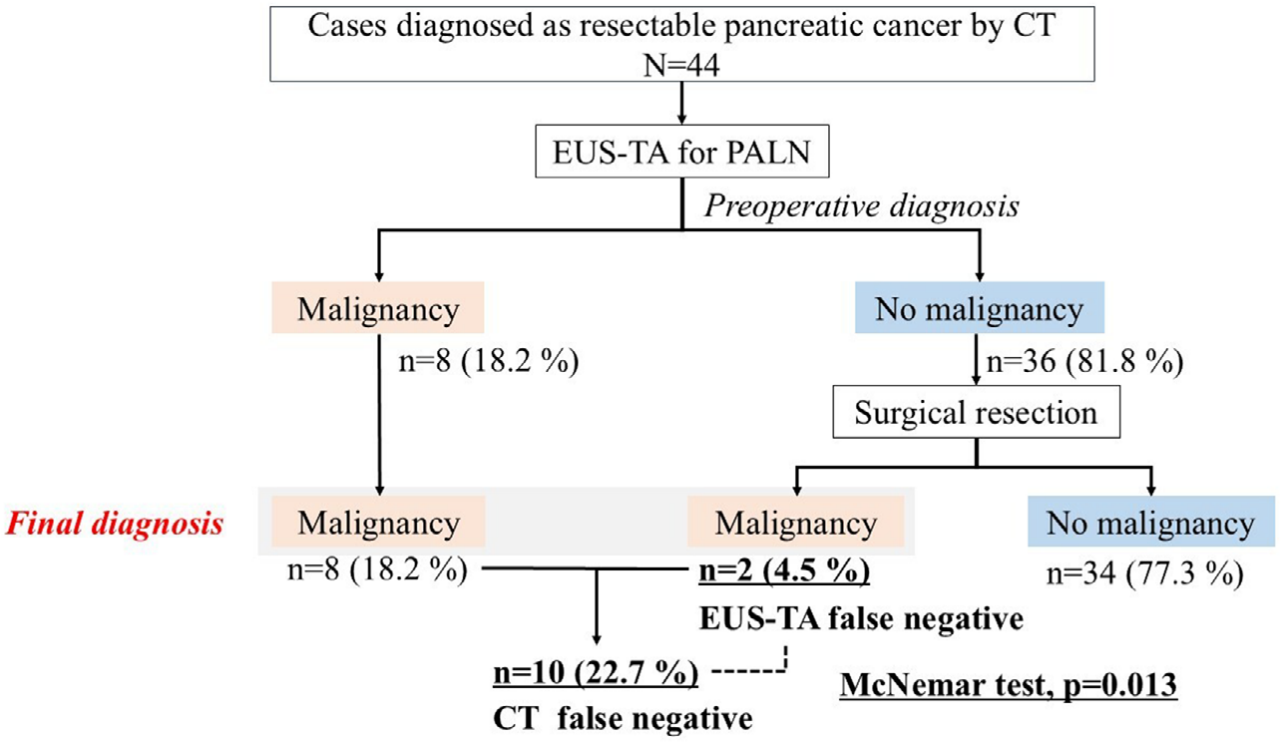
Clinical significance of endoscopic ultrasound‐guided tissue acquisition (EUS‐TA) for para‐aortic lymph node (PALN) in resectable pancreatic cancer by computed tomography (CT).

In all, 44 patients were diagnosed with resectable PC by CT. The outcomes of EUS‐TA for PALN metastasis in patients with resectable PC are shown in Figure 7. EUS‐TA identified PALN metastasis preoperatively in eight patients, diagnosing unresectable PC. Meanwhile, 36 patients were metastasis‐negative by EUS‐TA and underwent planned curative resection. The final diagnosis from the examination of the surgical specimens showed malignancy in two patients and no malignancy in 34. Based on CT alone, 10 patients would have undergone unnecessary surgery. Performing EUS‐TA for PALNs, compared with relying on CT diagnoses, resulted in significant avoidance of unnecessary surgery in eight patients (*P* = 0.013). The diagnostic capability of EUS‐TA for CT‐diagnosed resectable PC showed a sensitivity of 80.0% (8/10), a specificity of 100% (34/34), and an accuracy of 95.5% (42/44) (Table 4).

## DISCUSSION

Para‐aortic lymph node metastasis is commonly diagnosed using CT, primarily using PALN diameter for diagnosis.^9^, ^13^, ^14^, ^15^, ^16^ However, in this study the diagnostic accuracy of CT was only 64.8%, consistent with the findings of previous studies showing low accuracy for CT alone.^1^, ^2^, ^3^ Only one report used EUS‐TA for PALN metastasis, comparing its diagnostic capabilities with PET‐CT.^8^ We compared the diagnostic performance of PET‐CT and EUS‐TA. Although EUS‐TA is an invasive procedure, it demonstrated high accuracy and proved to be useful even in small PALNs or cases where CT or PET‐CT failed to diagnose metastases accurately. This study demonstrated that EUS‐TA for PALNs has a high technical success rate (97.0%) and accuracy (93.2%), significantly superior to CT. We identified priority targets for puncture by analyzing diagnostic performance based on the PALN size. For PALNs measuring 5–10 mm, the accuracy of CT was 51.5%, while EUS‐TA achieved a significantly higher accuracy (92.9%). EUS‐TA consistently demonstrated high accuracy (~90%) across all PALN sizes, underscoring its utility even in cases where CT alone fails to provide a definitive diagnosis. Additionally, among 44 patients with resectable PC by CT findings, 10 patients had PALN metastases. EUS‐TA successfully identified PALN metastases in eight patients preoperatively, enabling the avoidance of unnecessary surgeries. This highlights the role of EUS‐TA as a valuable modality for diagnosing PALN metastases that are undetectable by CT, thereby preventing unnecessary surgeries in preoperative settings. To our knowledge, this study represents the largest cohort demonstrating the clinical performance of EUS‐TA for PALNs and provides important clinical insights.

Although EUS‐TA is widely used for tissue acquisition,^17^, ^18^, ^19^, ^20^, ^21^, ^22^ reports on its performance, specifically for detecting PALN metastasis, remain limited, partly due to the difficulty in visualizing and puncturing PALNs. Our study showed low adverse events (0.6%), comparable to the general performance of EUS‐TA, underscoring its feasibility and safety for diagnosing PALN metastasis.^23^, ^24^, ^25^, ^26^ Therefore, EUS‐TA should be recommended for diagnosing PALN metastasis.

For intra‐abdominal lymph nodes, FNB has shown superior diagnostic capabilities than FNA, with some reports recommending their use.^27^, ^28^, ^29^ However, our results showed no significant difference in accuracy between FNA and FNB. The ability to avoid major blood vessels and puncture small PALNs with the more controllable FNA may explain their improved performance.

To determine which cases should be more aggressively targeted for PALN puncture, we analyzed the accuracy by size (Table 7). For PALNs measuring <5 mm and ≥10 mm, CT showed high accuracy, with no significant difference compared with EUS‐TA. For PALNs measuring 5–10 mm, EUS‐TA had a higher accuracy than CT. This analysis indicates that CT diagnosis based on PALN size tends to have a particularly low accuracy for PALNs measuring 5–10 mm. In contrast, EUS‐TA maintains a high diagnostic accuracy, regardless of the size. Therefore, puncture should be considered in preoperative cases where PALN visualization is possible, especially for PALNs measuring 5–10 mm, for which CT tends to be less effective.

In the analysis of false‐negative PALN cases, 63.6% of cases with PALN measured 5–10 mm. The utility of contrast‐enhanced EUS for lymph node metastasis evaluation has also been reported. For cases where EUS findings suggest metastasis, further investigations such as contrast‐enhanced EUS or repeat EUS‐TA should be considered to reduce false negatives.^10^, ^30^

In the three resected cases, tumors were primarily localized at the lymph node margins and distributed patchily. Early‐phase lymph node metastasis often involves only part of the node, particularly at the margins, potentially causing false‐negative results.^31^, ^32^ As EUS‐TA samples only a portion of the node, puncturing the entire node may improve early metastasis detection. For EUS‐TA targeting PALNs, using torque and fanning techniques during EUS‐TA may enhance tissue sampling and further improve accuracy.^33^, ^34^

Among CT‐diagnosed resectable PC, EUS‐TA identified PALN metastases in eight patients, rendering them unresectable. This preoperative identification helped avoid unnecessary surgeries in these patients. Without EUS‐TA, there was a potential for unnecessary surgery in 10 patients based on CT alone. However, EUS‐TA significantly reduced this risk, as surgery was performed in only two patients (*P* = 0.013). This result suggests that EUS‐TA has a significant clinical impact and can substantially alter treatment plans.

This study has limitations. First, its retrospective, single‐center study may limit generalizability. Second, some diagnoses were based on clinical course rather than pathological confirmation. Third, the preoperative waiting period was not considered in surgical cases, potentially influencing false‐negative cases due to disease progression. Fourth, the number of cases in which PET‐CT was performed was limited, potentially reducing the power of our analysis. Fifth, while contrast‐enhanced EUS could potentially improve the diagnosis of PALN metastases, further studies are warranted. Nonetheless, this study is unique for examining the largest cohort of patients with PALN metastasis undergoing EUS‐TA, and for assessing resectable PC and PALN size by CT.

In conclusion, EUS‐TA is a valuable modality for detecting PALN metastases that are not detectable by CT. Its accurate diagnostic capability makes it an effective tool for guiding treatment decisions and improving patient outcomes, especially when conventional imaging is inconclusive.

## CONFLICT OF INTEREST

Authors declare no conflict of interest for this article.

## FUNDING INFORMATION

This work was supported by The National Cancer Center Research and Development Fund (grant number 2022‐A‐16).

## ETHICS STATEMENT

Approval of the research protocol by an Institutional Reviewer Board: This study was approved by the Ethics Committee of the National Cancer Center (approval number: 2018–149).

Informed Consent: Written informed consent was obtained from all patients.

Registry and the Registration No. of the study/trial: N/A.

Animal Studies: N/A.

## Supporting information


**Table S1** Details of the puncture needle.
**Table S2** Comparison of endoscopic ultrasound‐guided tissue acquisition (EUS‐TA) and positron emission tomography‐computed tomography (PET‐CT).
**Table S3** Accuracy of computed tomography (CT), positron emission tomography (PET)‐CT, and endoscopic ultrasound‐guided tissue acquisition (EUS‐TA) in measuring the short axis diameter of para‐aortic lymph nodes (PALNs) Pub.
