# Supplementary material for: Clinical significance and efficacy of endoscopic ultrasound‐guided tissue acquisition for para‐aortic lymph node metastasis
Source: Dig Endosc. 2025 Mar 10;37(7):775–86. doi: 10.1111/den.15009 (PMC12244301; doi:10.1111/den.15009)
Supplement: Supplementary file 1 — Table S1 Details of the puncture needle. Table S2 Comparison of endoscopic ultrasound‐guided tissue acquisition (EUS‐TA) and positron emission tomography‐computed tomography (PET‐CT). Table S3 Accuracy of computed tomography (CT), positron emission tomography (PET)‐CT, and endoscopic ultrasound‐guided tissue acquisition (EUS‐TA) in measuring the short axis diameter of para‐aortic lymph nodes (PALNs) Pub. [file DEN-37-775-s001.docx]

**Supplementary Table 1.** Details of the puncture needle^†^

| **Puncture needle** | **Gauge** | **Patient (N=162)** |
| --- | --- | --- |
| FNA needle; EZ Shot 3 Plus (Olympus, Tokyo, Japan) | 22-gauge | 120 (74.1) |
| FNB needle; SharkCore (Medtronic Corporation, Newton, MA) | 22-gauge | 16 (9.9) |
| FNB needle; SonoTip TopGain (Medi-Globe, Achenmuhle, Germany) | 22-gauge | 13 (8.0) |
| FNB needle; Acquire (Boston Scientific Corporation, Massachusetts, USA) | 22-gauge | 5 (3.1) |
| Other needles | - | 8 (4.9) |

^†^Values are expressed as percentage.

FNA, fine-needle aspiration; FNB, fine-needle biopsy.

**Supplementary Table 2.** Comparison of EUS-TA and PET-CT^†^

|  | **Diagnostic yield ％ (95％CI）** | | |
| --- | --- | --- | --- |
|  | **PET-CT (n=32)** | **EUS-TA (n=32)** | **p-value*** |
| Sensitivity | 82.4% (56.6–96.2) | 76.5% (50.1–93.2) | 1.000 |
| Specificity | 66.7% (38.4–88.2) | 100% (69.8–100) | 0.734 |
| Positive predictive value | 73.7% (48.8–90.9) | 100% (66.1–100) | - |
| Negative predictive value | 76.9% (46.2–95.0) | 78.9% (54.4–93.9) | - |
| Accuracy | 75.0% (56.6–88.5) | 87.5% (71.0–96.5) | 0.343 |

^†^Values are presented as percentage (95% CI).

CI, confidence interval; EUS-TA, endoscopic ultrasound-guided tissue acquisition; PET-CT, positron emission tomography-computed tomography.

* McNemar test p-value.

**Supplementary Table 3.** Accuracy of CT, PET-CT, and EUS-TA in measuring the short axis diameter of PALNs^†^

| **Short axis diameter**  **of PALN (mm)** | **Malignancy rate** | **Accuracy ％ (95％CI）** | | |  |
| --- | --- | --- | --- | --- | --- |
|  |  | **CT (n=32)** | **PET-CT (n=32)** | **EUS-TA (n=32)** | **p-value*** |
| <5 | 0% (0/3) | 100% (29.2–100) | 33.3% (0.8–90.6) | 100% (29.2–100) | 0.135 |
| 5–10 | 54.5% (12/22) | 45.5% (24.4–67.8) | 77.3% (54.6–92.2) | 81.8% (59.7-94.8) | **<0.001** |
| ≥10 | 71.4% (5/7) | 71.4% (29.0–96.3) | 85.7% (42.1–99.6) | 100% (59.0–100) | 0.223 |
| Total | 53.1% (17/32) | 56.2% (37.7–73.6) | 75.0% (56.6–88.5) | 87.5% (71.0–96.5) | **<0.001** |

^†^Values are presented as percentage (95% CI).

CI, confidence interval; CT, computed tomography; EUS-TA, endoscopic ultrasound-guided tissue acquisition; PET-CT, positron emission tomography-computed tomography.

* Cochran Q test p-value.
